# Central retinal artery occlusion in a child with ADA2 deficiency: a case report

**DOI:** 10.1097/MS9.0000000000001857

**Published:** 2024-02-28

**Authors:** Israa Sharabati, Baraa M. Ayesh, Ruaa Mustafa Qafesha, Heba Rasras, Fawzy M. Abunejma, Mohammed Abdulrazzak, Afnan W.M. Jobran

**Affiliations:** a Faculty of Medicine, Al Quds University, Jerusalem; b Ahli Hospital, PRCS Hebron Hospital, Hebron University, Hebron, Palestine; c Faculty of Medicine, University of Aleppo, Aleppo, Syria

**Keywords:** ADA2 deficiency, ADA2 mutation, case report, central retinal artery occlusion

## Abstract

**Introduction and importance:**

Deficiency of ADA2 (DADA2) is the first molecularly described monogenic vasculitis syndrome. During the past decade, DADA2’s clinical spectrum has expanded significantly as the number of reported cases has increased.

**Case presentation:**

A 5-year-old boy with DADA2 who experienced sudden onset left-sided vision loss due to unilateral central retinal artery occlusion. The patient had a history of recurrent fever and arthralgia with high inflammatory markers (C-reactive protein and erythrocyte sedimentation rate). Brain MRI showed mild limbic encephalitis, and MRA was normal. His gene sequencing results demonstrated substitutions mutation in ADA2, and the diagnosis of DADA2 was eventually confirmed.

**Clinical discussion:**

Central retinal artery occlusion (CRAO) in paediatrics is a very rare condition. Typically, DADA2 presents in childhood as systemic inflammation, vasculitis, humoral immunodeficiency, and/or haematologic abnormalities. The most common phenotype described in the literature is vasculitis, which typically affects the skin and central nervous system, but other systems can also be affected. Ophthalmic manifestations are less common and highly variable.

**Conclusions:**

DADA2 manifests rarely with central retinal artery occlusion; therefore, physicians should be aware of this manifestation.

## Introduction and importance

Highlights

- Deficiency of ADA2 is an inherited systemic autoinflammatory disorder caused by ADA2 gene mutation.
- A previously healthy 3-year-old boy was presented to the emergency room due to a sudden, left-sided loss of vision with a severe headache.
- Central retinal artery occlusion (CRAO) in paediatrics is a very rare condition.
- The patient is stable on weekly etanercept injections.

Central retinal artery occlusion (CRAO) in paediatrics is a very rare condition. Approximately one case occurs every 50 000 people younger than 30. The most common causes of retinal artery occlusions in children are trauma, migraine headaches, and cardiac emboli. Other possible causes include Inflammatory conditions, coagulation abnormalities, and intraocular diseases^1^. For DADA2, however, CRAO is a rare symptom: only eight cases have been reported in the literature^2–5^.

Deficiency of ADA2 (DADA2) is the first molecularly described monogenic vasculitis syndrome. Deficiency of ADA2 DADA2 is an autosomal recessive autoinflammatory condition, first reported in 2014^2,6^, that manifests in three main categories of illness: vasculitis/vasculopathy, immunodeficiency, and haematological disease. During the past decade, DADA2’s clinical spectrum has expanded significantly as the number of reported cases has increased^7^. It is common for DADA2 to manifest as systemic inflammation or elevated acute phase reactants, which makes early diagnosis more difficult^1^. The diagnosis should be reevaluated in patients who are not responding or who exhibit unexplained symptoms.

In this study, we present a case of central retinal artery occlusion due to DADA2 in a 5-year-old male patient, highlighting the challenges in diagnosis and managing this case.

## Case presentation

**Figure 1:**
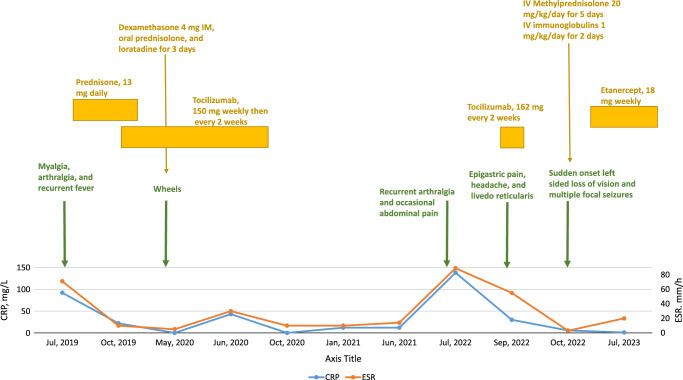
The clinical course of the patient. CRP, C-reactive protein; ESR, erythrocyte sedimentation rate.

A previously healthy 3-year-old boy was in his usual state of health until he developed lower limb pain. (Fig. 1) On examination, he had tender calf muscles. He was treated for mostly reactive myalgia with ibuprofen. The patient started to develop a recurrent fever for 3 weeks with 3–4 temperature spikes per day with a maximum temperature of 40°C. It was associated with bilateral knee joint pain that was entirely relieved by ibuprofen. No joint tenderness, effusion, or any other abnormal signs were noted on examination. Parents were second-degree relatives.

Blood tests revealed a haemoglobin count of 9.6 mg/l and a mean corpuscular volume of 64.9. The C-reactive protein (CRP) concentration was 65 mg/l, and the erythrocyte sedimentation rate (ESR) was 70 mm/h. Others, including white blood cells, platelets, serum electrolytes, liver enzymes, antistreptolysin antibodies, rheumatoid factor, antinuclear antibodies, the brucella test, cytomegalovirus and Ebstein-Barr virus serology, C3 and C4, and creatinine kinase, were unremarkable. The main laboratory findings throughout his illness are shown in Table S1.

Abdominal ultrasound showed splenomegaly (11.5 cm) with a homogenous pattern and a liver span of 10.5 cm. The whole-body computed tomography (CT) scan showed splenomegaly (10 cm) with no other related findings. Echocardiography, hip ultrasound, and bone marrow biopsy were unremarkable. A genetic study of common variants of familial mediterranean fever showed the heterozygous M694V mutation.

In this context of knee joint pain with recurrent fever and splenomegaly, it was suggested that he had systemic juvenile idiopathic arthritis. The ophthalmic examination didn’t show any abnormal findings. He was treated with steroids orally (13 mg daily) for 1 month, which didn’t resolve the high-grade fever, ESR, and CRP. So, he was started on tocilizumab injections (150 mg every week for 2 months, then 150 mg every 2 weeks).

While he was on tocilizumab, he developed a pruritic skin rash in the form of wheels with elevated ESR and CRP levels. He was given dexamethasone (4 mg IM) once, as well as prednisone and loratadine orally for 3 days. It was suggested that he had a relapse. So, he continued the treatment for another 8 months before it was stopped. He remained free of any complaints until July 2022.

The patient started suffering from multiple symptoms, beginning with periodic polyarthralgia and occasional abdominal pain. The labs showed high CRP and low haemoglobin levels. Two months later, because of a lack of improvement, he was started on a tocilizumab injection (162 mg every 2 weeks). After the second dose, the patient developed severe epigastric pain, which increased at night and was associated with headaches and livedo reticularis all over his extremities. It was suspected to be due to tocilizumab; therefore, it was discontinued.

Six weeks later, the patient presented to the emergency room due to a sudden, left-sided loss of vision with a severe headache. He developed right-sided convulsions and eye twitches that were aborted with intravenous diazepam (5 mg). He subsequently had one episode of fever at 38.5°C.

**Figure 2:**
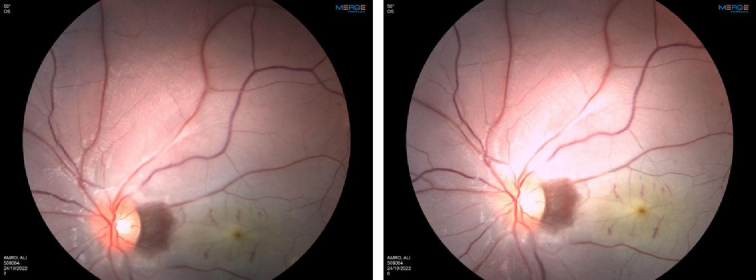
Fundoscopic examination of the left eye of the patient.

Ophthalmic examination revealed a cherry-red spot in the left eye (Fig. 2), and a central retinal artery occlusion was diagnosed. Complete blood count showed a haemoglobin of 10.6 (MCV 66), a white blood cell count of 6100 cells/µl and a platelet count of 218 000/ µl. Lumbar puncture did not reveal any abnormalities in the cerebrospinal fluid.

**Figure 3:**
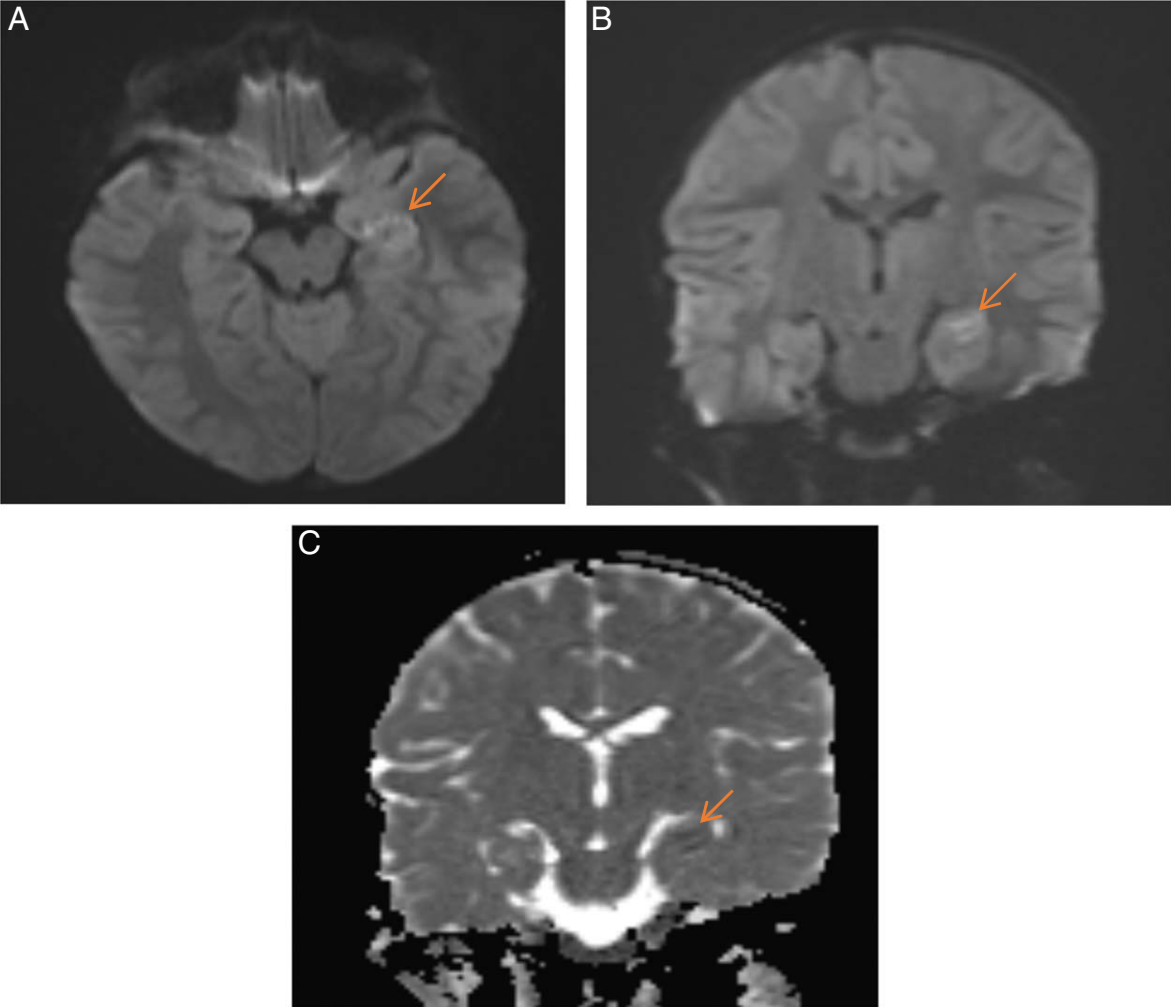
(A) Axial and (B) coronal view showing subtle abnormal signal intensity (abnormal hyperintensity) on the diffusion imaging in the medial left temporal lobe in the region of hippocampal formation. (C) Coronal view apparent diffusion coefficient (ADC) MRI, there is corresponding decrease signal on the ADC maps in this area. Mostly limbic encephalitis, there is no evidence of acute infraction or haemorrhage in the remainder of the imaging of the brain. The remainder of the MRI of the brain is within normal limits.

Brain MRI showed on diffusion-weighted imaging (DWI) increased intensity in the left medial temporal lobe and caudate nucleus in the region of the hippocampal formation, suggestive of mild limbic encephalitis (Fig. 3). Electroencephalogram, echocardiography, and carotid duplex were in the normal range and didn’t refer to any potential conditions. Magnetic resonance angiography and venography (MRA and MRV) did not show any pathology. Abdominal ultrasound revealed splenomegaly (a span of 13 cm) and mesenteric lymphadenitis.

He was treated with a five-day pulse of intravenous methylprednisolone (20 mg/kg/day) and intravenous immunoglobulins for 2 days (1 g/kg/day). Enoxaparin sodium (clexane) was administered subcutaneously (20 mg once daily), and he was also given aspirin tablets (80 mg daily). Neurologically, he improved without a motor or sensory deficit. While hospitalized, his blood pressure (BP) measurements were elevated (systolic BP range was 130–150 mmHg and diastolic range was 85–102 mmHg), so he was treated with IV hydralazine (4 mg every 4 h) and then switched to oral amlodipine (2.5 mg twice daily). Renal doppler ultrasound was normal.

Moreover, the results of a genetic study of genes associated with thrombophilia (e.g. Factor V leiden, B-fibrinogen, etc.) did not explain the patient’s symptoms. Prothrombin/activated partial thromboplastin time, serum lipids, rheumatoid factor, anticardiolipin, anti-glycoprotein 1 antibody, lupus anticoagulant, protein C, C3, C4, and serum homocysteine were found to be within normal limits. The ophthalmic exam revealed visual acuity in right eye 6/6, left eye 6/95 and the fundoscopic exam showed a left flat cherry-red spot.

We reassessed the diagnosis and performed whole-exome sequencing for suspected monogenic inherited autoinflammatory disorders. A homozygous A->G substitution at chr22:17687970 confirms the diagnosis of DADA2 (germline mutation of the ADA2/ CECR1 gene c.533T>C leading to p.Phe178Ser). Treatment with etanercept (18 mg weekly subcutaneous injections) was initiated.

Six months after diagnosis, the patient is stable on weekly etanercept injections. There was a partial improvement in his left eye’s visual acuity (6/38); a fundoscopic exam showed pale optic disc temporally with macular atrophy and he was advised to patch the right eye daily for 3–4 hours.

## Clinical discussion

DADA2 is an inherited systemic autoinflammatory disorder caused by ADA2 gene mutation which can be homozygous or compound heterozygous. ADA2 deficiency results in chronic exposure to adenosine, prolonged tissue inflammation occurs which causes tissue damage and fibrosis, as well as vascular inflammation and vasculopathy^7^. It is important to keep in mind that, although the same mutation is present, the vasculopathy in this illness differs in terms of organ involvement and severity, resulting in a wide variety of symptoms ranging from asymptomatic to life-threatening^8^.

Typically, DADA2 presents in childhood as systemic inflammation, vasculitis, humoral immunodeficiency, and/or haematologic abnormalities. The most common phenotype described in the literature is vasculitis, which typically affects the skin and central nervous system, but other systems can also be affected^6^. Neurologic involvement presents mainly as recurrent strokes^7^. Other manifestations include peripheral neuropathy, cranial nerve palsy, and neurosensory hearing loss. Ophthalmic manifestations are less common and highly variable. Patients can have conjunctivitis, uveitis, papillitis, optic neuritis and sudden visual loss^9,10^. Zhou *et al*.^2^ reported the first case of central retinal artery occlusion (total of 9 patients). More recently, a second cohort study conducted in China reported five patients with CRAO, which coincide with our patient presentation. MRI findings of DADA2 are consistent with small-sized vasculopathy and lacunar infarctions/haemorrhages in the brainstem and deep brain regions. In another study of nine patients, brain MRIs revealed acute and/or chronic ischaemic lesions (75%); six had recurrent episodes of ischaemia^3^. Our patient, however, had limbic encephalitis on MRI. Both MRA and MRV were normal.

Skin involvement is reported in 90% of cases^11^. Half of patients with vasculitis cutaneous manifestations have a livedoid rash, as in our patient, and it can be the only clinical manifestation. Additionally, patients may develop cutaneous nodules, Raynaud’s phenomenon, ulcers, and digital gangrene. Our patient also had systemic inflammation and symptoms such as fever, arthralgia and morning stiffness, elevated CRP and ESR^7^.

Moreover, DADA2 patients frequently develop abdominal and gastrointestinal manifestations, including recurrent abdominal pain, hepatosplenomegaly, and intestinal perforation, as seen in our patient who initially had abdominal pain and splenomegaly. These symptoms are due to vasculopathy that affecting the superficial and deep abdominal arteries^12,13^.

Together with the patient’s medical history and his new symptoms, we considered genetic testing. Whole-exome sequencing result showed homozygous mutation of the ADA2 gene c.533T>C leading to p.Phe178Ser, 44 previous cases with DADA2 were reported before with this mutation (supplementary file, Supplemental Digital Content 1, http://links.lww.com/MS9/A387). In general, the use of genetic sequencing is crucial for diagnosis, but other methods can be used such as measurement of ADA2 enzymatic activity in the serum^7^. Each test serves a specific purpose in ruling out other potential conditions and providing valuable information for diagnosis and management. The normal findings from the carotid duplex, EEG, and echocardiography tests are significant as they help exclude specific conditions and guide further evaluation to determine the underlying cause of the patient’s symptoms following tocilizumab treatment. By elucidating the rationale behind conducting these tests and emphasizing their role in ruling out other potential conditions. the case report provides a comprehensive assessment of the patient’s clinical presentation and supports a thorough diagnostic approach to optimize patient care and outcomes^6^.

Biologic treatments play an essential role in managing cases with DADA2 disease. Anti-TNF drugs like etanercept, adalimumab, and infliximab is the drug of choice for DADA2, even after the failure of immunosuppressive treatment. it improves the overall clinical manifestations, control fever episodes and vasculopathy, and prevent strokes in patients with ADA2 deficiency syndrome. Although steroids and other immunosuppressive drugs can be used, they are thought not to prevent strokes^14^. In this case, the disease was well controlled on Adalimumab. Moreover, TNF inhibitors were effective in treating patients that have focal segmental glomerulonephritis in addition to DADA2 with plasmapheresis, and are strongly recommended as a first-line therapy to treat vasculitis^15^.

## Conclusions

patients presenting with features of CRAO require a thorough systemic evaluation and complete haematological and genetic workup. As seen in our patient, uncommon conditions such as DADA2, which can manifest in a very wide range of clinical presentations, could be the underlying cause. With prompt diagnosis and treatment, other serious manifestations such as stroke could be prevented. This case focuses on the importance of a systemic workup in patients with CRAO.

## Ethical approval

Not applicable.

## Informed consent

Written informed consent was obtained from the patient’s family for reporting this case and its associated images. The consent is available for review on request.

## Source of funding

Not applicable.

## Author contribution

All authors fulfil the authorship criteria because of their substantial contributions to the conception, design, analysis, and interpretation of the data. I.S. and H.R. administrated the project, analyzed the data and drafted the manuscript. I.S. and H.R. participated in data acquisition and participated in the manuscript drafting. R.M.Q. and B.M.A. participated in the manuscript drafting and find resources. All authors reviewed the study and wrote the final version. F.M.A. participated in data acquisition, and participated in its design. M.A. reviewed the study and participated in writing the final version. A.M.W.J. conceived the study, and participated in its design. All authors read and approved the final manuscript.

## Conflicts of interest disclosure

The authors declare that they have no competing interests.

## Research registration unique identifying number (UIN)

Our research study does not involve human subjects.

## Guarantor

Afnan W. M. Jobran.

## Data availability statement

Not applicable.

## Provenance and peer review

Not commissioned, externally peer-reviewed.

## Supplementary Material
