# Supplementary material for: Central retinal artery occlusion in a child with ADA2 deficiency: a case report
Source: Ann Med Surg (Lond). 2024 Feb 28;86(4):2343–7. doi: 10.1097/MS9.0000000000001857 (PMC10990381; doi:10.1097/MS9.0000000000001857)
Supplement: Supplementary file 1 [file ms9-86-2343-s001.docx]

| ESR | CRP | Platelets | WBC | RBC | Hemoglobin | Date and events |
| --- | --- | --- | --- | --- | --- | --- |
| 70 | 56 | 427 | 8.8 | 4.5 | 9.6 | 13^th^-Jul-2019. |
|  | 106 | 384 | 7 | 4.11 | 8.8 | 15^th^-Jul-2019 |
| 71 | 92 | 347 | 6.7 | 4.5 | 9 | 18^th^-Jul-2019 |
| 73 | 48 | - | - | - | - | 23^rd^-Jul-2019 |
| 62 | 30 | 478 | 8.5 | 4.55 | 9 | 28^th^-Jul-2019 |
| 75 | 101 | 393 | 6.7 | 3.8 | 7.7 | 4^th^-Aug-2019. |
| 30 | 12 | 619 | 12.4 | 4.55 | 9.7 | 17^th^-Aug-2019 on steroids |
| 30 | 36 | 510 | 13.7 | 4.4 | 9.3 | 20^th^-Aug-2019 |
| 28 | 18 | 432 | 14.2 | 4.5 | 9.5 | **27^th^-Aug-2019 started on steroids** |
| 20 | 16 | 436 | 8.8 | 4.8 | 9.6 | 7^th^-Sep-2019 |
| 29 | 24 | 381 | 6.6 | 4.6 | 9.3 | **17^th^-Sep-2019, started on Tocilizumab** |
| - | - | 385 | 6.1 | 5.4 | 10 | 4t-Oct-2019 |
| 5 | 8 | 304 | 8.99 | 5.88 | 10.4 | 21^st^-Oct-2019 |
| 35 | 164 | 360 | 8 | 4.99 | 9.2 | 2^nd^-Oct-2019 |
| 10 | 22 | 234 | 11.7 | 5 | 9.7 | 16^th^-Oct-2019 |
| 10 | 34 | 231 | 9.2 | 5.2 | 10 | 30t-Nov-2019 |
| - | neg | 249 | 10.3 | 5.5 | 10.4 | 14^th^-Dec-2019 |
| - | 10 | 260 | 6.9 | 5.11 | 10.3 | 28^th^-Dec-2019 |
| - | 25 | 149 | 2.75 | 5 | 10.3 | 11^th^-Jan-2020 |
| - | neg | 239 | 3.32 | 5 | 10.6 | 25^th^-Jan-2020 |
| - | neg | 182 | 3.13 | 4.6 | 10.1 | 8^th^-Feb-2020 |
| - | 28 | 251 | 4.23 | 4.75 | 11.7 | 22^nd^-Feb-2020 |
| - | neg | 264 | 5.28 | 4.58 | 10.5 | 14^th^-Mar-2020 |
| - | neg | 217 | 3.63 | 5 | 11.4 | 2^nd^-Apr-2020 |
| 5 | neg | 252 | 6.2 | 5.05 | 12.1 | 25th-May-20220 |
| 30 | 43 | 309 | 4.8 | 4.75 | 11.2 | 6^th^-Jun-2020 |
| 18 | neg | 342 | 4.6 | 4.3 | 10.8 | 12^th^-Jul-2020 |
| 17 | 6 | 339 | 5.1 | 4.7 | 11.3 | 27^th^-Aug-2020 |
| 10 | neg | 309 | 4.3 | 4.5 | 11 | 14^th^-Oct-2020 |
| 17 | 12 | 230 | 4.7 | 4.4 | 10.5 | 10^th^-Dec-2020 |
| 10 | 12 | 297 | 4.2 | 4.7 | 11.1 | 9^th^-Jan-2021 |
| 14 | 12 | 375 | 4.9 | 4.86 | 10.7 | 21^st^-Jun-2021 |
| 89 | 137.9 | 401 | 7.1 | 4.4 | 9.8 | 23^rd^-Jul-2022 |
| 71 | 24 | 340 | 6.2 | 4.4 | 8.8 | 3^rd^-Aug-2022 |
| 58 first hour | 24 | 286 | 4.9 | 4.5 | 8.9 | 9^th^-Aug-2022 |
| 53 | 24 | 322 | 4.9 | 4.5 | 9.4 | 7^th^-Sep-2022 |
| 55 | 30 | 211 | 4.3 |  | 11 | **14^th^-Sep-2022, restarted** on Tocilizumab |
| 3 | 5 | 282 | 5.2 | 4.8 | 10.9 | 20^th^-Oct-2022 |
| 3 | 5.5 | 410 | 21.3 | 5.77 | 13.1 | **23^rd^-Oct-2022. Admission to Hospital due to CRAO and seizure** |
| - | 21 | 249 | 17.6 | 5.34 | 12 | 24^th^-Oct-2022 |
| - | - | 254 | 4.6 | 4.77 | 10.6 | 26^th^-Oct-2022 |
| - | 3.9 | 262 | 4.9 | 5 | 10.6 | 27^th^-Oct-2022 |
| - | 1.6 | 182 | 10.2 | 5.4 | 12.3 | 29^th^-Oct-2022 |
| - | - | 245 | 5.9 | 5.2 | 12.5 | 21^st^-Dec-2022 |
| - | - | 253 | 4.9 | 4.8 | 12.2 | 28^th^-Jan-2023 |
| - | - | 228 | 4.2 | 5 | 12.2 | 4^th^-Feb-2023 |
| - | - | 325 | 6.2 | 4.98 | 12.2 | 18^th^-Feb-2023 |
|  |  |  |  |  |  | **3-2023, Diagnosed with ADA2 Deficiency, started on Etanercept** |
| 20 | 1 | 270 | 3.9 | 4.7 | 12.5 | 24^th^-Jul-2023 |

**Table S1:** The main laboratory findings throughout the illness.
